# Evaluation of the Oral Health-Related Quality of Life (OHRQoL) in Patients Undergoing Lingual Versus Labial Fixed Orthodontic Appliances: A Randomized Controlled Clinical Trial

**DOI:** 10.7759/cureus.23379

**Published:** 2022-03-22

**Authors:** Jehad M Kara-Boulad, Ahmad S Burhan, Mohammad Y Hajeer, Tarek Z. Khattab, Fehmieh R Nawaya

**Affiliations:** 1 Department of Orthodontics, University of Damascus Faculty of Dentistry, Damascus, SYR; 2 Department of Orthodontics, University of Hama Faculty of Dentistry, Hama, SYR; 3 Department of Pediatric Dentistry, Syrian Private University Faculty of Dentistry, Damascus, SYR

**Keywords:** psychological disability, physical disability, psychological discomfort, physical pain, functional limitation, ohip-14, quality of life, oral health impact profile-14, lingual appliance, labial appliance

## Abstract

Background

Wearing fixed orthodontic appliances may negatively impact oral health-related quality of life (OHRQoL) during treatment. This study aimed to compare the OHRQoL of patients treated with labial or lingual appliances.

Methodology

A total of 38 patients (23 females, 15 males; mean age: 21.3 years) with class I malocclusion and moderate crowding in the upper and lower dental arches were included. These patients were planned to be treated on a non-extraction basis and were randomly divided into the following two groups: the lingual appliance (LA) group and the buccal appliance (BA) group. The Oral Health Impact Profile-14 (OHIP-14) questionnaire was used to measure the OHRQoL at the following six assessment times: before treatment (T0), one week after treatment (T1), one month after treatment (T2), three months after treatment (T3), six months after treatment (T4), and at the end of the active treatment (T5).

Results

In total, 19 patients in each group were included in the final analysis with no dropouts. In both groups, the overall OHIP-14 scores increased and peaked on the first week following appliance placement and then significantly decreased over time. The LA group had significantly greater overall OHIP‑14 scores than the labial group at T1 (p < 0.001) and T2 (p = 0.004) only.

Conclusions

The OHRQoL improved in both lingual and labial groups after treatment. Moreover, it was better in the labial group compared to the lingual group during the first month of treatment. In both groups, the greatest deterioration in OHRQoL occurred in the first week and gradually decreased over time.

## Introduction

Most adult patients are concerned about the appearance of orthodontic appliances, which impacts their physical, social, and psychological well-being. Presently, orthodontics aims to achieve patients’ desired improved appearance and aesthetics for orthodontic appliances [1]. Labial brackets may cause unaesthetic appearance and functional restrictions [2]. Lingual appliances have started to provide a viable alternative for the successful treatment of most adult and adolescent patients. These appliances meet patients’ desires for a complete aesthetic appliance and allow the orthodontist to fully control the movement of the teeth in three dimensions [3].

The main goal of orthodontic treatment is to balance aesthetic, functional, and patients’ ambitions, which contributes to their quality of life [4]. Several factors affect the quality of life during orthodontic treatment, such as pain, difficulty eating, change of speech, and diet changes [5]. Recently, several published papers have focused on the assessment of patient-oriented outcome measures following different treatment modalities, such as removable appliances in interceptive orthodontics [6], bone-anchored intermaxillary traction [7], functional appliances [8], rapid and slow maxillary expansion appliances [9], the accelerated fixed orthodontic treatments [10,11].

The concept of oral health-related quality of life (OHRQoL) was introduced to describe the assessment of an individual’s well-being on several domains, including pain, discomfort, psychological function, physical function, and social function. Thus, it helps us understand how malocclusion can affect people’s lives [12]. Many questionnaires have been used to measure OHRQoL, with the most widely used and comprehensive one being the Oral Health Impact Profile (OHIP). The OHIP questionnaire originally consisted of 49 questions (OHIP-49), but Slade shortened it to 14 items (OHIP-14) because the original questionnaire took a long time to complete [13]. Researchers and clinicians prefer the OHIP-14 to the OHIP-49 because of its practicability, reliability, and validity [14]. Researchers recommend the quality of life assessment in orthodontics to plan treatment, evaluate outcomes and therapy efficiency, understand patients’ expectations, and help them cope with the consequences of the treatment [15].

Few studies evaluating the effect of orthodontic treatment on the OHRQoL have shown, due to functional limitations, an improvement in the OHRQoL after treatment and a decrease in the OHRQoL during the first few weeks of treatment [16].

Lingual brackets cause speech disturbances, tongue irritation, and difficulty in chewing, with more social embarrassment than labial brackets [17,18]. Several studies have investigated the OHRQoL during orthodontic treatment with fixed labial appliances [19-21]. The majority of studies that have compared lingual against labial appliances have focused on adverse effects, such as pain, discomfort, chewing disturbances, speech impairment, and oral hygiene problems [17,22,23], whereas others have focused on biomechanical aspects [24]. Only one study evaluated the OHRQoL among the labial, lingual, low-friction brackets, and Invisalign® appliances [25]. However, this study had several shortcomings. The assessment of OHRQoL was conducted only on one occasion at one month following the beginning of the treatment; therefore, the pre-treatment status, as well as the changes that occurred following appliance wear, were not evaluated. Second, pain and discomfort were only assessed at several time points in the first week of treatment only, which did not reflect the whole picture of patients’ response to the given appliances. Third, the age range of recruited patients was too large (i.e., between 18 and 40 years). Finally, the four groups at baseline assessment were statistically different in terms of age and sex distribution which sheds doubts on the validity of the comparisons.

However, the intensity and extent of the impact caused by lingual appliances on the OHRQoL compared to that caused by labial appliances are not yet clear. There is no published randomized controlled trial comparing labial and lingual fixed orthodontic treatment in terms of OHRQoL. Therefore, the objective of the current trial was to evaluate the effects of using labial versus lingual orthodontic appliances on the OHRQoL employing the OHIP-14 questionnaire on a group of patients with class I malocclusion and moderate crowding.

## Materials and methods

Trial design and registration

This study was a parallel-group randomized controlled trial. The study was reviewed and approved by the Local Research Ethics Committee at the University of Damascus Dental School (UDDS-260-06012018/SRC-2620). It was registered at ClinicalTrials.gov (Reference number: NCT03850951) and was funded by the University of Damascus Postgraduate Research Budget (Reference number: 83063206771DEN). No changes in the methodology occurred after the beginning of the trial.

Sample size calculation

The sample size was calculated using Minitab™ 17 software (Minitab Inc., State College, PA, USA) with a two-sample t-test, a selected study power of 0.80 to identify a two-point difference in OHIP-14 overall score, a significance level of 0.05, and an estimated standard deviation (SD) of 2.1 according to a previous study [25]. The analysis revealed that 19 patients were required for each group.

Participants and eligibility criteria

**Figure 1:**
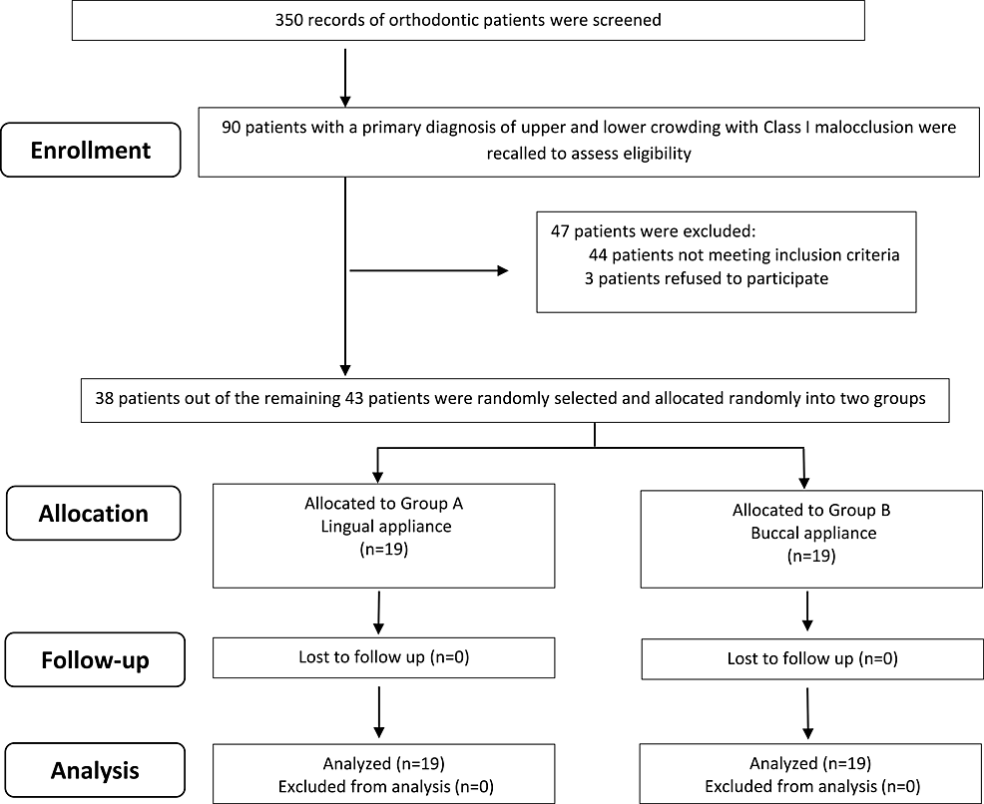
CONSORT flow diagram of participants’ recruitment, follow-up, and entry into data analysis.

A total of 90 patients registered at the Department of Orthodontics at the University of Damascus Dental School with a primary diagnosis of crowded teeth and class 1 malocclusion were recalled for further examination. The inclusion criteria included: (1) Class I molar canine relationships on both sides; (2) moderate crowding of both the arches of about 4 to 6 mm that could be treated on a non-extraction basis; (3) age from 18 to 25 years; (4) no anterior crossbites; and (5) no craniofacial syndromes, cleft lip, and/or cleft palate (soft and/or hard). Thirty-eight patients (23 females, 15 males; mean age: 21.3 years) who met the inclusion criteria were randomly selected and divided into two equal-sized groups. The treatment protocol was explained to the patients before participating in the study. All patients were informed that participation was voluntary, and upon their acceptance, they were asked for their written consent. A CONSORT flow diagram of participants’ recruitment, follow-up, and entry into data analysis is shown in Figure 1.

Randomization, allocation concealment, and blinding

Randomization was performed using a computer-generated random list (Excel 2007, Microsoft Windows, Microsoft, Chicago, IL, USA) with an allocation ratio of 1:1. The patients were randomly allocated into the two groups using sealed and sequentially numbered envelopes. The allocation procedure was concealed from the researcher and was conducted by one of the co-authors. Blinding of both patients and the main researcher was not possible due to the visibility of the appliances. Blindness was only applied during data analysis.

First group: the lingual fixed orthodontic group (LA)

**Figure 2:**
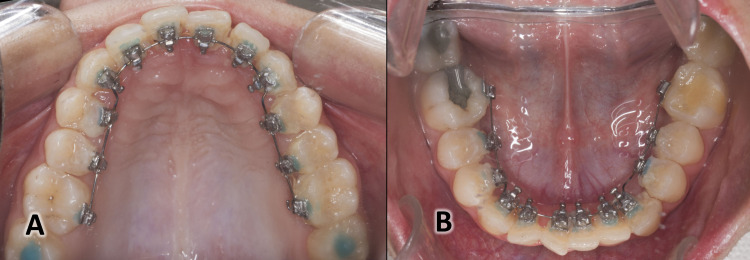
The lingual appliance used in the current study. (A) An occlusal view of the lingual appliance in the upper jaw. (B) An occlusal view of the lingual appliance in the lower jaw.

The brackets with 0.018-inch slots (DTC Orthodontics, Hangzhou, China) were applied with the aid of a special indirect bonding technique “the HIRO System” [26]. The lingual brackets were placed on both arches at the same appointment (Figure 2). Individual lingual archwires (Forestadent®, Germany) were fabricated directly on the initial dental cast using an Arch Forming Turret (Dentaurum Inc. Langhorne, USA) with a prominence premolar offset only. The archwires sequence was 0.012", 0.014", 0.016" nickel-titanium, 0.016" × 0.022" TMA, 0.016" × 0.022" SS, and 0.017" × 0.025" SS.

Second group: the buccal fixed orthodontic group (BA)

The brackets with 0.018-inch slots (American Orthodontics brackets, Mini Master series, MBT prescription) were used and directly bonded on both arches. Prefabricated archwires (American Orthodontics, Sheboygan, WI, USA) were used in the following sequence 0.012"-0.014"-0.016" nickel-titanium, 0.016" × 0.022" nickel-titanium, 0.016" × 0.022", and 0.017" × 0.022" SS.

In both groups, the initial archwire was applied immediately after applying the appliances, and patients were followed up. Archwires were only replaced when there was an improvement in teeth alignment and the following archwire could be inserted with a minimal amount of bending and without exerting excessive force on teeth [27]. A gentle interproximal reduction was carried out from canine to canine to create the required space for aligning the crowded teeth according to the requirements of each case using single-sided, hand-held metal abrasion strips (Steelcarbo® strips, Horico, Berlin, Germany). The treatment was finished with 0.014" stainless steel archwire for occlusal stability and to give some detail where required.

Evaluation of oral health-related quality of life

OHIP-14 questionnaire was used to measure the OHRQoL during treatment. It consisted of 14 items covering seven domains: functional limitation, physical pain, psychological discomfort, physical disability, psychological disability, social disability, and handicap [14]. Because the mother tongue of the recruited patients in the current trial was Arabic, the Arabic version of the OHIP‑14 questionnaire was used which has been shown to have high reliability and validity [28].

After inserting the appliance, patients had to complete an OHIP‑14 questionnaire to analyze the impact of the used appliances on their OHRQoL. This questionnaire was given to patients at six assessment times: before treatment (T0), one week after treatment (T1), one month after treatment (T2), three months after treatment (T3), six months after treatment (T4), and at the end of the treatment (T5). The response to each item was scored on a five-point Likert scale (0 = never, 1 = almost never, 2 = occasionally, 3 = quite often, 4 = very often). The score 0 referred to a good quality of life whereas 5 referred to the worst. Scores ranged from 0 to 8 for each domain. The overall OHIP-14 scores ranged from 0 to 56 which were the sum of the seven domains. A higher OHIP-14 score indicated a worse QoL [14].

Data analysis

Data analysis was performed using SPSS version 22.0 (IBM Corp., Armonk, NY USA), and all data were blinded and transferred to an Excel spreadsheet software (Microsoft, Redmond, WA, USA). Because of normal distribution, two-sample t-tests were employed to examine intergroup differences. Paired sample t-tests were used to fulfill the multiple comparisons to evaluate intragroup changes. Statistical significance was set at the 0.05 level.

## Results

All patients completed the different assessment times with no withdrawals. Thus, a response rate of 100% was achieved. The basic characteristics of the study sample are presented in Table 1.

**Table 1:** Basic sample characteristics in terms of gender distribution, age, and treatment time. Two sample t-test was used for comparisons made in age and treatment time, whereas the chi-square test was used for the analysis of gender distribution between the two groups. The level of significance was set at 0.05. SD: standard deviation

Variable	Lingual group	Labial group	P-value
Gender (females/males)	10/9	13/6	0.194
Mean age ± SD (years)	21.7 ± 3	20.8 ± 2.8	0.389
Mean treatment time ± SD (years) (months)	13.8 ± 2.1	14.3 ± 2.6	0.881

Before brackets’ placement (T0), the mean of overall OHIP-14 scores was 9.74 and 9.37 for the LA and BA groups, respectively. In both groups, the overall OHIP scores significantly increased (p ˂ 0.001) in comparison with the T0 values and peaked on the first week following treatment: 27 and 19.84 for the LA and BA groups, respectively. The mean scores decreased significantly over time. At the final assessment time (T5), mean scores in both groups reached values smaller than what was recorded at baseline (T0), that is, 3.53 and 3.37 for the LA and BA groups, respectively (p ˂ 0.001) (Table 2). Patients in the LA group had significantly greater mean overall OHIP‑14 scores than those in the BA group at one week following the onset of treatment (T1; p < 0.001) and at one month (T2; p = 0.004). However, there were no significant differences between the two groups at T0, T3, T4, and T5 (p > 0.05) (Table 3).

**Table 2:** Descriptive statistics of the OHIP-14 scores for each group at all assessment times as well as the p-values of significance testing against the baseline scores (T0). Paired t-tests were used for intragroup comparisons (i.e., pairwise comparisons against the T0 scores). *Significant at p < 0.05. SD: standard deviation; LA: lingual appliance; BA: buccal appliance; T0: before treatment; T1: one week after treatment; T2: one month after treatment; T3: three months after treatment; T4: six months after treatment; T5: at the end of the treatment; OHIP-14: Oral Health Impact Profile-14

Domain	Time	Lingual appliance			Buccal appliance		
		Mean	SD	P-value (vs. T0)	Mean	SD	P-value (vs. T0)
Functional limitation	T0	0.11	0.32		0.11	0.32	
	T0-T1	5.21	1.03	<0.001*	2.21	0.98	<0.001*
	T0-T2	3.26	1.15	<0.001*	0.89	0.81	<0.001*
	T0-T3	1.42	0.77	<0.001*	0.26	0.45	0.187
	T0-T4	0.58	0.51	0.003*	0.21	0.42	0.331
	T0-T5	0.53	0.51	0.007*	0.16	0.37	0.578
Physical pain	T0	0.42	0.61		0.42	0.77	
	T0-T1	5.95	0.71	<0.001*	4.16	1.07	<0.001*
	T0-T2	3.68	0.95	<0.001*	2.37	1.01	<0.001*
	T0-T3	2.21	0.85	<0.001*	1.21	0.54	0.002*
	T0-T4	0.89	0.46	0.008*	0.53	0.51	0.650
	T0-T5	0.68	0.48	0.096	0.32	0.48	0.650
Psychological discomfort	T0	2.05	2.15		1.58	1.35	
	T0-T1	3.32	1.60	<0.001*	3.53	0.96	<0.001*
	T0-T2	1.68	1.06	0.340	1.79	0.71	0.331
	T0-T3	0.84	0.69	0.009*	0.89	0.74	0.033*
	T0-T4	0.63	0.60	0.003*	0.58	0.61	0.001*
	T0-T5	0.37	0.50	0.002*	0.37	0.60	0.000*
Physical disability	T0	0.79	1.18		0.74	0.93	
	T0-T1	4.63	0.96	<0.001*	3.53	1.02	<0.001*
	T0-T2	2.47	1.07	<0.001*	1.58	0.90	0.003*
	T0-T3	1.42	0.84	0.036*	0.58	0.51	0.420
	T0-T4	0.84	0.50	0.853	0.53	0.51	0.297
	T0-T5	0.63	0.60	0.563	0.53	0.51	0.297
Psychological disability	T0	2.16	1.61		2.16	0.96	
	T0-T1	3.37	1.34	<0.001*	3.53	0.77	<0.001*
	T0-T2	1.37	0.76	0.015*	3.05	0.78	<0.001*
	T0-T3	0.58	0.51	<0.001*	2.32	0.75	0.482
	T0-T4	0.42	0.51	<0.001*	1.63	0.50	0.021*
	T0-T5	0.42	0.51	<0.001*	1.37	0.68	0.007*
Social disability	T0	2.42	2.09		2.16	1.42	
	T0-T1	3.42	1.84	0.008*	1.68	1.06	0.120
	T0-T2	1.79	1.03	0.111	0.89	0.66	0.004*
	T0-T3	0.79	0.54	0.001*	0.68	0.67	0.001*
	T0-T4	0.58	0.51	0.001*	0.47	0.51	<0.001*
	T0-T5	0.47	0.51	<0.001*	0.32	0.48	<0.001*
Handicap	T0	1.79	1.78		2.32	2.08	
	T0-T1	1.11	1.10	0.028*	1.21	1.23	<0.001*
	T0-T2	0.58	0.69	0.004*	0.47	0.84	<0.001*
	T0-T3	0.42	0.51	0.001*	0.42	0.77	<0.001*
	T0-T4	0.42	0.51	0.002*	0.37	0.68	<0.001*
	T0-T5	0.42	0.51	0.001*	0.32	0.58	<0.001*
Total OHIP-14	T0	9.74	6.24		9.37	4.18	
	T0-T1	27.00	4.89	<0.001*	19.84	3.99	<0.001*
	T0-T2	14.84	4.37	<0.001*	11.05	3.08	0.018*
	T0-T3	7.68	3.13	0.060	6.37	2.54	<0.001*
	T0-T4	4.37	1.83	<0.001*	4.32	2.00	<0.001*
	T0-T5	3.53	2.06	<0.001*	3.37	1.80	<0.001*

**Table 3:** Comparison of OHIP-14 scores between the two groups at all assessment times as well as the p-values of significance testing. Two sample t-tests were conducted for the comparisons between the two groups. *Significant at p < 0.05. SD: standard deviation; LA: lingual appliance; BA: buccal appliance; T0: before treatment; T1: one week after treatment; T2: one month after treatment; T3: three months after treatment; T4: six months after treatment; T5: at the end of the treatment; OHIP-14: Oral Health Impact Profile-14

Domain	Group	T0		T1		T2		T3		T4		T5	
		Mean	P-value	Mean	P-value	Mean	P-value	Mean	P-value	Mean	P-value	Mean	P-value
Functional limitation (Items 1 + 2)	LA	0.11	1.000	5.21	<0.001*	3.26	<0.001*	1.42	<0.001*	0.58	0.020*	0.53	0.016*
	BA	0.11		2.21		0.89		0.26		0.21		0.16	
Physical pain (Items 3 + 4)	LA	0.42	1.000	5.95	<0.001*	3.68	<0.001*	2.21	<0.001*	0.89	0.025*	0.68	0.023*
	BA	0.42		4.16		2.37		1.21		0.53		0.32	
Psychological discomfort (Items 5 + 6)	LA	2.05	0.421	3.32	0.626	1.68	0.721	0.84	0.821	0.63	0.789	0.37	1.000
	BA	1.58		3.53		1.79		0.89		0.58		0.37	
Physical disability (Items 7 + 8)	LA	0.79	0.880	4.63	0.001*	2.47	0.009*	1.42	0.001*	0.84	0.063	0.63	0.564
	BA	0.74		3.53		1.58		0.58		0.53		0.53	
Psychological disability (Items 9 + 10)	LA	2.16	1.000	3.37	0.659	1.37	<0.001*	0.58	<0.001*	0.42	<0.001*	0.42	<0.001*
	BA	2.16		3.53		3.05		2.32		1.63		1.37	
Social disability (Items 11 + 12)	LA	2.42	0.653	3.42	0.001*	1.79	0.003*	0.79	0.596	0.58	0.529	0.47	0.333
	BA	2.16		1.68		0.89		0.68		0.47		0.32	
Handicap (Items 13 + 14)	LA	1.79	0.408	1.11	0.782	0.58	0.676	0.42	1.000	0.42	0.789	0.42	0.556
	BA	2.32		1.21		0.47		0.42		0.37		0.32	
Overall OHIP-14 score	LA	9.74	0.832	27.00	<0.001*	14.84	0.004*	7.68	0.163	4.37	0.933	3.53	0.803
	BA	9.37		19.84		11.05		6.37		4.32		3.37	

Functional limitation and physical pain significantly increased (p < 0.001) and peaked on the first week following treatment in both groups and then decreased over time; nevertheless, the scores were still significant in the LA group at all assessment times for functional limitation and up to T4 for physical pain in comparison with T0 values (p ˂ 0.05) (Table 2). Scores of functional limitation and physical pain were significantly greater in the LA group compared to the BA group at all assessment times (Table 3).

Physical disability significantly increased at T1, T2, and T3 (p < 0.05) in the LA group, and at T1 and T2 (p < 0.05) in the BA group in comparison with T0 values. The highest scores were recorded at T1, that is, 4.63 and 3.53 for the LA and the BA groups, respectively. The LA group had a significantly greater physical disability than what was recorded in the BA group at T1, T2, and T3 (p < 0.05) (Table 3).

Psychological disability significantly increased at the first week following treatment (T1; p < 0.001) in the LA group, while significant increases were recorded at one week following the onset of treatment and at one month (p < 0.001) in the BA group in comparison with T0 values (Table 2). The BA group had a significantly greater psychological disability than those in the LA group at T2, T3, T4, T5 (p < 0.05) (Table 3).

For psychological discomfort, social disability, and handicap, there were no significant differences between the two groups (p ˃ 0.05) at all assessment times, except for social disability at one week following treatment (T1) and at one month (T2) where a statistically significant difference between the two groups was observed (p < 0.05), with the LA group having higher mean scores in comparison to those in the BA group (Table 3). There were significant differences between the assessment times when intergroup comparisons were conducted (p ˂ 0.001) (Table 2).

## Discussion

There is little published data on the effect of the lingual appliance on OHRQoL, even though wearing this appliance may affect physical, emotional, and psychological aspects of life. To our knowledge, this is the first randomized controlled trial that measures and compares OHRQoL between lingual and labial fixed appliances at different time points during orthodontic treatment. In this study, the OHIP-14 questionnaire was chosen to measure OHRQoL because it is the most widely used in the literature and can be easily filled by patients [14]. Al‐Jundi et al. (2007) validated the Arabic version of this questionnaire among Arabic-speaking adult patients [28].

In this study, the OHRQoL improved significantly in both groups after treatment. This improvement could be explained by the enhancement in the position of the teeth after the leveling and alignment stage. This is in agreement with previous studies which found that patient satisfaction and OHRQoL improved at the end of orthodontic treatment [20,29,30]. Our study found that the greatest deterioration in OHRQoL occurred in the first week and gradually decreased over time, which could be attributed to the adaptation of the patient or to the experience gained. This finding is in agreement with the results of Chen et al. (2010) who reported that the OHRQoL was the worst after one week of the insertion of fixed appliances. Moreover, they reported that this was because the combination of physical pain, psychological discomfort, and physical disability were at the highest levels [15].

This study showed that the ORHQoL of the labial group was better than that of the lingual group during the first month without significant differences from the third month to the end of treatment. These results do not agree with the results of Antonio-Zancajo et al. (2020) who found that the lingual bracket had the least negative impact on their OHRQoL compared with the conventional and low-friction brackets and aligners, with statistically significant differences in pain, psychological discomfort, psychological disability, social disability, and total OHIP-14 score [25]. This difference may be due to the type of lingual brackets used because Antonio-Zancajo et al. applied the STB brackets which were smaller and more comfortable than the ones used in the current trial.

The functional limitation and physical pain in this study were significantly greater in the lingual group compared to the labial group at all assessment times. As previously discussed, lingual appliances are more associated with speech disturbances than labial ones, and patients with lingual appliances are more likely to report a perception of articulation changes [31]. This finding does not agree with those of Antonio-Zancajo et al. who did not find significant differences between conventional and lingual appliances regarding functional limitation [25]. Additionally, they found that patients with lingual brackets encountered lower levels of pain than those undergoing fixed labial brackets. Another study by Sadek et al. found insignificant differences in functional limitation and physical pain between labial or lingual appliances [32]. However, Sadek et al. investigated the lingual bio-creative therapy technique which uses a flat lingual retractor bonded to the lingual surface of the anterior teeth, which may have caused less discomfort than what was reported in this study using conventional lingual brackets.

The results of the current work revealed that physical disability was significantly greater in the lingual group compared to the labial group. This can be explained by the presence of bite planes, which produce a posterior disocclusion, and by the restriction of the tongue space due to the close proximity of the tongue to the lingual brackets leading to swallowing difficulties particularly during the early phase of the treatment until the reflex of swallowing was relearned. This finding agreed with those of Wu et al. [23] and Madurantakam and Kumar [33] who found that patients treated with lingual appliances experienced more swallowing difficulties and dietary changes than those treated with labial appliances [23,33]. Our finding disagrees with the results of Binhuwaishel and Al-Jewair [34] who found that eating difficulty was not significantly different between lingual and labial appliances [34]. This may be due to the study design, mean ages of the participants, and the types of questions asked. Our study results are in agreement with those of Khattab et al. [17] who found that both lingual and labial appliances caused chewing difficulty; however, lingual appliances had significantly higher scores of impairment compared to labial appliances [17].

Our study findings showed that psychological disability was significantly greater in the labial group compared to the lingual group at T2, T3, T4, and T5. Lingual appliances are considered ultimate esthetic appliances and are of special importance for patients’ physical appearances, as well as their psychological and social lives [35]. This agreed with Sadek et al. who found that patients treated with labial bio-creative therapy were more annoyed by the appearance of the appliance and were more likely to be embarrassed compared with those treated with the lingual bio-creative technique [32]. Antonio-Zancajo et al. found that patients with conventional brackets reported a greater negative impact than patients with lingual brackets regarding psychological discomfort and psychological disability [25].

Social disability was significantly greater in the lingual group compared to the labial group at T1 and T2 without differences in handicap. This agrees with the findings of Wu et al. [23] and Sadek et al. [32] who reported that patients treated with lingual appliances reported more adverse social impacts than those treated with labial appliances [23,32]. Our results are also in agreement with those of Antonio-Zancajo et al. who did not find statistically significant differences regarding handicap between conventional and lingual appliances [25].

Limitations

The evaluation of patients’ OHRQoL using their subjective assessment on the OHIP-14 questionnaire can be considered one of the limitations of this trial. Their responses on the questionnaire could be affected by several factors, such as their compliance and cooperation, their honesty during answering the questions, their emotional state at the time of giving answers, and the accuracy and attention being paid. In this study, a traditional lingual bracket system was used, but other lingual orthodontic systems may have a different impact on the patients’ OHRQoL. Furthermore, this study did not evaluate gender differences which requires a larger sample size.

## Conclusions

The OHRQoL improved in both lingual and labial groups after treatment. It was better in the labial group compared to the lingual group during the first month of treatment. In both groups, the greatest deterioration in OHRQoL occurred in the first week and gradually decreased over time. Functional limitation, physical pain, physical disability, and social disability were greater in the lingual group compared to the labial group. Psychological discomfort and handicap were not significantly different between the two groups. Psychological disability was significantly greater in the labial group compared to the lingual group.
